# The habenular nuclei: a conserved asymmetric relay station in the vertebrate brain

**DOI:** 10.1098/rstb.2008.0213

**Published:** 2008-12-04

**Authors:** Isaac H. Bianco, Stephen W. Wilson

**Affiliations:** 1 Department of Cell and Developmental Biology, University College LondonLondon WC1E 6BT, UK; 2 Department of Molecular and Cellular Biology, Harvard UniversityCambridge, MA 02138, USA

**Keywords:** habenula, asymmetry, interpeduncular nucleus, zebrafish, dorsal diencephalic conduction system

## Abstract

The dorsal diencephalon, or epithalamus, contains the bilaterally paired habenular nuclei and the pineal complex. The habenulae form part of the dorsal diencephalic conduction (DDC) system, a highly conserved pathway found in all vertebrates. In this review, we shall describe the neuroanatomy of the DDC, consider its physiology and behavioural involvement, and discuss examples of neural asymmetries within both habenular circuitry and the pineal complex. We will discuss studies in zebrafish, which have examined the organization and development of this circuit, uncovered how asymmetry is represented at the level of individual neurons and determined how such left–right differences arise during development.

## 1. Anatomy and connectivity

The dorsal diencephalic conduction (DDC) system is one of two major pathways that interconnect the limbic forebrain and sites in the mid- and hindbrain, the other pathway being the medial forebrain bundle (MFB; Sutherland 1982). These two pathways appear to represent parallel neural circuits—they share sources of afferent inputs as well as efferent targets and there is an overlap in their physiology and function.

The DDC comprises three core components: the habenular nuclei; the stria medullaris (SM), which is the main fibre tract through which inputs from the forebrain arrive at the habenulae; and the fasciculus retroflexus (FR), a prominent fibre tract that predominantly carries efferent axons from the habenula towards the targets in the midbrain/hindbrain. In this review, we will focus on the anatomy and connectivity of the habenulae and the interpeduncular nucleus (IPN). The latter is a major target of habenular efferent connectivity in all vertebrates and consequently plays a pivotal role in the modulation of nuclei downstream of the DDC. In §3, we will review the various classes of neural asymmetry that have been described in the DDC, especially of anamniotes, and in §4, we will discuss work in zebrafish that has addressed the developmental mechanisms by which DDC circuit asymmetries emerge.

### (a) Habenula

The bilaterally paired habenular nuclei (Hb) are positioned adjacent to the third ventricle, rostral to the posterior commissure and the habenular commissure runs between them (Nieuwenhuys *et al*. 1998; Butler & Hodos 2005).

**Figure 1:**
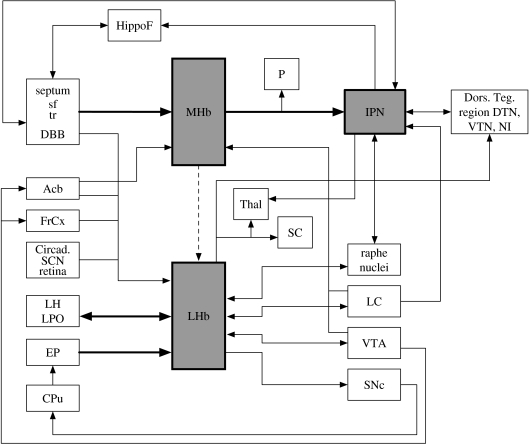
Connectivity of the DDC. This schematic shows the principal connections of the medial and lateral habenulae and interpeduncular nucleus as described in mammals, in particular the rat. Thick arrows highlight the septum–MHb–IPN axis and the convergence of limbic and striatal inputs into the lateral habenula. Notably, there are very limited data regarding the relative functional importance of the various connections shown here. Acb, nucleus accumbens; Circad., potential sources of circadian information; CPu, caudate/putamen; Dors. Teg. region, dorsal tegmental region; DBB, nucleus of diagonal band; DTN, ventral tegmental nucleus of Güdden; EP, entopeduncular nucleus; FrCx, frontal cortex; HippoF, hippocampal formation; IPN, interpeduncular nucleus; LC, locus coeruleus; LH, lateral hypothalamic area; LHb, lateral habenula; LPO, lateral preoptic area; MHb, medial habenula; NI, nucleus incerta; P, pineal; SC, superior colliculus; SCN, suprachiasmatic nucleus; SNc, substantia nigra pars compacta; sf, septofimbrial nucleus; Thal, thalamic nuclei; tr, nucleus triangularis; VTA, ventral tegmental area; VTN, ventral tegmental nucleus of Güdden.

In mammals, the habenular complex comprises two separate nuclei on each side: the ‘medial’ (MHb) and ‘lateral’ (LHb) habenulae. The LHb is further subdivided into principal medial and lateral subdivisions. Despite sharing some sources of afferent inputs and efferent targets, the medial and lateral habenulae appear to represent largely distinct subcircuits within the DDC (Herkenham & Nauta 1977, 1979; Kim & Chang 2005). In outlining the anatomy of this circuit, we shall focus primarily on the patterns of connectivity in the rat, which have been well studied (figure 1). Some species differences will also be mentioned, where relevant, but we will not attempt a description of the comparative neuroanatomy of the DDC in any detail.

#### (i) Medial habenula

MHb circuitry is highly conserved (Sutherland 1982). The MHb primarily receives inputs from the septum and projects to the IPN of the ventral midbrain. This efferent connection comprises the ‘core’ of the FR and appears to be conserved in all vertebrate species that have been examined.

##### Afferent connectivity

The major source of afferent innervation of the MHb derives from the supracommissural septum, with axons coursing in the SM (Herkenham & Nauta 1977). Septal sites themselves receive inputs from the hippocampus and subiculum. Axons from the two most significant septal nuclei also terminate in different subdomains of the MHb—the septofimbrial nucleus innervates the rostral MHb while the nucleus triangularis innervates the caudal MHb. In the rat, almost every neuron in these two septal nuclei is likely to project to the MHb. More minor inputs derive from the nucleus of the diagonal band (DBB).

The MHb also receives ascending inputs, principally derived from monoaminergic nuclei, which are also the targets of both medial and lateral habenular efferent axons. Dopaminergic inputs derive from the interfascicular nucleus of the ventral tegmental area (VTA; Phillipson & Pycock 1982) and noradrenergic inputs from the locus coeruleus (Gottesfeld 1983). These latter axons reach the MHb by coursing anteriorly in the MFB and then joining the SM.

##### Efferent connectivity

The main target of MHb axons is the IPN (Herkenham & Nauta 1979). The MHb contains both cholinergic neurons (in its ventral two-thirds) and dorsally located substance P-containing neurons (Contestabile *et al*. 1987). Both types are contacted by the major afferent axons from the septofimbrial nucleus and nucleus triangularis, and both project down the core of the FR to innervate the IPN. MHb axons terminate in a topographic manner, wherein the neurons of the dorsal MHb innervate the lateral IPN, those of the medial MHb innervate the ventral IPN and lateral MHb neurons project to the dorsal IPN (Herkenham & Nauta 1979; Contestabile & Flumerfelt 1981).

After making lesions in the MHb, Ronnekleiv & Moller (1979) observed degenerating terminals in the pineal organ, suggesting that it may also be an MHb efferent target.

Although little is known about the intrinsic habenular circuitry and the extent to which communication exists between the MHb and LHb, two observations provide evidence for a medial-to-lateral connection. A subset of MHb axons project through the LHb and in so doing display *en passant* boutons that might represent presynaptic terminals (Kim & Chang 2005), and sectioning of the MHb efferent axons has been reported to reduce substance P levels in the LHb (see Sutherland 1982).

#### (ii) Lateral habenula

When compared with the MHb, the LHb shows broader and less evolutionarily conserved connectivity. It is thought to be involved in the motor–limbic interface because it receives significant pallidal inputs as well as afferent connections from numerous components of the limbic system. The LHb projects to a wide range of targets, especially in the ventral midbrain tegmentum (Sutherland 1982).

##### Afferent connectivity

The LHb is a point of convergence for neural information from the basal ganglia and limbic forebrain.

A major source of innervation of the LHb in the rat derives from the entopeduncular nucleus (EP, which is the non-primate equivalent of the internal segment of the globus pallidus). In the rat, virtually every entopeduncular neuron appears to project to the LHb, suggesting that the axons are collaterals of the pallido-thalamic pathway (Herkenham & Nauta 1977). This pallido-habenular pathway also exists in cats and monkeys. However, in monkeys, it appears that while the LHb receives substantial innervation from the internal segment of the globus pallidus, this innervation derives from a different group of pallidal neurons to those that innervate premotor neurons in the thalamus and brainstem (Parent *et al*. 2001).

Limbic regions of the forebrain constitute the second major source of afferent innervation of the LHb. A continuous band of cells, stretching from the anterior lateral preoptic area, through the lateral hypothalamus, to the mid-hypothalamus, project to the LHb (Herkenham & Nauta 1977). A small descending input derives from septal regions, including the DBB and lateral septal nucleus. Additionally, the LHb receives inputs from the nucleus accumbens and medial frontal cortex (Greatrex & Phillipson 1982).

The suprachiasmatic nucleus, which is concerned with the generation of circadian rhythms in mammals, projects vasopressin-containing axons to the LHb (Buijs 1978; Sofroniew & Weindl 1978). A second source of circadian information is suggested by the finding that in mice, melanopsin-expressing retinal ganglion cells project to the LHb (Hattar *et al*. 2006).

The LHb receives ascending innervation from monoaminergic nuclei, at least some of which overlap with the sources innervating the MHb. Thus, axons from the median raphe and locus coeruleus provide serotonergic and noradrenergic inputs (Herkenham & Nauta 1977; Gottesfeld 1983; Vertes *et al*. 1999). Midline neurons of the VTA (interfascicular and paranigral nuclei) provide dopaminergic inputs to the medial part of the LHb, probably via the FR (Phillipson & Pycock 1982; Gruber *et al*. 2007). Notably, this region of the VTA contains many neurons belonging to the A10 region, which gives rise to the mesolimbic ‘reward’ pathway.

In summary, the afferent connectivity of the LHb may enable motivational/emotional states (encoded by limbic inputs) to modulate motor behaviours (represented by pallidal efferents).

##### Efferent connectivity

The LHb contains predominantly glutamatergic neurons as well as some GABAergic and cholinergic cells and establishes efferent connectivity with a wide range of targets. Many of these targets are themselves sources of afferent inputs to the LHb (Herkenham & Nauta 1979; Lecourtier & Kelly 2007). For example, the LHb projects to the limbic forebrain, including the lateral hypothalamic area, lateral preoptic area, substantia innominata and ventrolateral septum.

The LHb establishes descending connectivity with numerous monoaminergic nuclei in the mid- and hindbrain. A major projection, especially from the medial LHb, innervates the median and dorsal raphe; LHb activity inhibits the raphe (Wang & Aghajanian 1977), probably as a result of activation of GABAergic interneurons in the nucleus (e.g. Varga *et al*. 2003). The LHb innervates and inhibits the dopaminergic VTA (Araki *et al*. 1988) and the substantia nigra *pars compacta* (SNc; see below, §2). There are several routes for feedback in this circuit: the VTA projects directly to the LHb and also to the nucleus accumbens, which is a source of LHb afferent innervation. Dopaminergic neurons of the SNc project, via the nigrostriatal tract, to the dorsal striatum (caudate/putamen), which in turn connects to the pallidum, a major source of afferent innervation of the LHb. The connectivity of the DDC therefore enables the striatum to regulate the activity of midbrain dopaminergic (DA) neurons that provide its afferent inputs; Sasaki *et al*. (1990) have provided functional data supporting a role for the EP, SM and habenula in negative feedback control over the SNc.

Other efferent targets of the LHb include several thalamic nuclei (centromedial, mediodorsal, ventromedial, parafascicular nucleus), the superior colliculus, the dorsal tegmental region and locus coeruleus (Herkenham & Nauta 1979).

### (b) Interpeduncular nucleus

The IPN receives most of the efferent axons from the MHb, and therefore is central to MHb control over downstream circuitry.

The IPN is a singular, unpaired structure, located at the ventral midline of the posterior midbrain/isthmus. It comprises a number of morphologically defined subnuclei. In the rat, Lenn & Hamill (1984) have identified seven subnuclei, including three that are described as unpaired, being located at the midline and which are flanked laterally by four bilaterally paired subnuclei. In addition to the MHb, the IPN is interconnected with numerous sites in the forebrain and brainstem, and a multitude of neurotransmitters are expressed in a spatially organized manner within its subnuclei. These features suggest that the IPN is an important integrative centre and relay station within the limbic system (see Morley 1986).

#### (i) Afferent connectivity

A major source of innervation of the IPN is from the MHb, a connectivity pattern that is conserved throughout the vertebrate lineage (Shibata *et al*. 1986; Butler & Hodos 2005). Afferent inputs derive from several other sites within the limbic forebrain, which in the rat include the medial frontal cortex, DBB, substantia innominata, preoptic and hypothalamic nuclei and the supramammillary nucleus. In addition, various nuclei within the brainstem project to the IPN including the raphe, locus coeruleus and dorsal tegmental region (including the dorsal tegmental nucleus of Güdden, laterodorsal tegmental nucleus and nucleus incerta; Contestabile & Flumerfelt 1981; Hamill & Jacobowitz 1984; Shibata *et al*. 1986; Vertes & Fass 1988; Takagishi & Chiba 1991).

Biochemical studies have identified extremely high levels of acetylcholine, choline acetyltransferase, acetylcholine esterase and high-affinity choline uptake within the IPN, and the habenulo-interpeduncular pathway is considered one of the major cholinergic pathways in the brain (Contestabile & Fonnum 1983). Cholinergic innervation is likely to derive from both the MHb and the dorsal tegmental region and neurons in the basal forebrain (septum and preoptic area); the latter are thought to project the axons that extend, uninterrupted, through the habenula and FR to reach the IPN (Contestabile & Fonnum 1983; Woolf & Butcher 1985). There is evidence that a wide range of additional neurotransmitters are present in the IPN, including γ-aminobutyric acid (GABA, probably deriving from the DBB), substance P (from the MHb) and various monoamines (noradrenaline, dopamine and serotonin) and neuropeptides (including cholecystokinin, leucine-encephalin, methionine-encephalin, vasointestinal peptide and somatostatin) (see Morley 1986).

As mentioned above, MHb axons terminate in a topographic manner within the IPN, and accordingly, Contestabile *et al*. (1987) and Eckenrode *et al*. (1987) have shown that cholinergic and substance P-containing inputs are largely segregated within the IPN. Cholinergic fibres are confined in the unpaired midline core of the IPN, whereas substance P signalling shows greater localization to the peripheral subnuclei.

#### (ii) Efferent connectivity

The dorsal tegmental region and the raphe are the major targets of IPN efferent connectivity (Shibata & Suzuki 1984). In addition, the IPN makes ascending projections to various neuronal nuclei, several of which are the sources of afferent inputs to the habenulae, thus establishing further feedback circuits. Thus, efferent targets in the forebrain include the DBB and the lateral septal nucleus, preoptic area, dorsolateral hypothalamus, mediodorsal nucleus of the thalamus and the hippocampus and entorhinal cortex (Shibata & Suzuki 1984; Groenewegen *et al*. 1986; Morley 1986; Vertes & Fass 1988).

## 2. Physiological and behavioural functions

In accordance with the diversity of its afferent inputs and efferent targets, the DDC is involved in a diverse range of cerebral functions (previously reviewed in Sutherland 1982; Klemm 2004; Lecourtier & Kelly 2007). One central theme is the importance of the DDC (especially the habenular nuclei) in regulating the activity of monoaminergic nuclei in the ventral midbrain.

### (a) Control of dopaminergic circuitry: motor activity and reward prediction

Several recent reports provide evidence for strong functional links between the LHb and dopaminergic cells in the ventral midbrain, which are involved in modulating motor behaviours and learning new behavioural responses to salient stimuli.

Habenular lesions increase exploratory behaviour and locomotor activity in rats, especially in response to novel environmental stimuli (Lee & Huang 1988). This effect is likely to be mediated by midbrain DA neurons that are innervated by the LHb. Electrical stimulation of the LHb inhibits the activity of DA neurons in the VTA and SNc (Christoph *et al*. 1986), probably as a result of excitatory LHb efferents activating GABAergic interneurons in the ventral midbrain, which in turn inhibit DA cells (Ji & Shepard 2007). Conversely, habenular lesions result in increased dopaminergic transmission (Lisoprawski *et al*. 1980; Nishikawa *et al*. 1986), suggesting that habenular efferent circuitry exerts a tonic inhibitory effect upon DA neurons. Lecourtier *et al*. (2008) have suggested that within the VTA, neurons involved in reward seeking, which project to the nucleus accumbens, are preferentially inhibited. Suppressing LHb activity with a GABA antagonist causes increases in DA release in the Acb and striatum of similar timecourse and magnitude to those observed during reward-seeking behaviour (but causes a significantly smaller increase in the prefrontal cortex). The elevated DA levels in the nucleus accumbens (Acb) and striatum correlated with increases in spontaneous locomotor activity and stereotyped behaviours (grooming, sniffing, digging and rearing), respectively.

Short ‘phasic’ responses of midbrain DA neurons are thought to provide teaching signals that modulate the selection of motor programmes in the striatum and are involved in appetitive learning of new behavioural responses to positive reinforcers (‘rewards’; Schultz 1998). The LHb is one of the few regions of the brain to be inhibited by hedonic stimuli (Gallistel *et al*. 1985), and recently, Matsumoto & Hikosaka (2007) have shown that the LHb instructs midbrain DA neurons as to the absence of a reward. In monkeys performing a visually guided saccade task, LHb neurons are activated by visual targets that signify the absence of a reward and inhibited by targets that predict forthcoming reward (whereas DA neurons of the SNc show the opposite responsiveness). In unrewarded trials, the activation of LHb neurons preceded the inhibition of SNc neurons and mild electrical stimulation of the LHb inhibits the SNc. Therefore, it appears that the LHb provides information regarding the nature of salient environmental stimuli to the midbrain reward circuits in the form of negative reward-related signals. Thus, it is likely to be involved in the adjustment of behavioural strategies. Notably, functional magnetic resonance imaging (fMRI) has shown that the habenula of human subjects is activated in response to the informative negative feedback that indicates behavioural errors and also when positive feedback following correct responses is omitted (Ullsperger & von Cramon 2003). It is likely, therefore, that DDC circuitry is also involved in reward processing in humans, wherein Hb activation is expected to reduce the probability of phasic DA activation in the VTA and SNc.

### (b) Cognition

The DDC has been implicated in cognitive processes, in particular relating to spatial learning and attention.

An involvement of the habenular complex in spatial learning is suggested by the studies in rats using the classical Morris water maze test. Villarreal *et al*. (2002) found that after training in the water maze, aged, memory-impaired rats showed reduced cytochrome oxidase activity (a read-out of neuronal activity) in the LHb when compared with young unimpaired rats. The LHb was one of only a few brain regions to show this change and it was not observed after control swimming in the absence of learning, suggesting that LHb metabolic activity is correlated with spatial memory performance. Supporting a functional role for the habenula in spatial reference memory, Lecourtier *et al*. (2004) showed that (medial and lateral) Hb lesions impaired memory acquisition and retrieval in the Morris water maze. Spatial memory involves the hippocampus, which also contributes the major afferent input to the septofimbrial nucleus and nucleus triangularis. These septal nuclei in turn provide the major input to the MHb (§1*a*). Therefore, the habenula might be involved in learning via the integration of hippocampal signals, relating to memory formation or retrieval, with the activity of the Acb, relating to whether a behavioural strategy has been rewarded (Lecourtier & Kelly 2007). Moreover, habenular lesions alter synaptic plasticity between the hippocampus and Acb (Lecourtier *et al*. 2006).

Experiments using a specific behavioural assay of attention in rats, the 5-choice serial reaction time test, implicated the habenulae in attentional mechanisms (Lecourtier & Kelly 2005). In this test, habenular lesions result in an increase in premature responding, which might represent the emergence of an impulsive mode of behaviour. The effect is blocked by haloperidol, suggesting that it is caused by increased dopaminergic transmission from the midbrain DA neurons, which are inhibited by the descending habenular efferents (above). A second effect is that rats display a progressive deterioration in choice accuracy, which is probably not due to dysregulation of DA signalling. While the mechanism of this defect is unclear, it might involve changes in noradrenergic or cholinergic transmission, both of which are affected by DDC circuitry (discussed in Lecourtier & Kelly 2007). The observation that choice accuracy is not impaired immediately after surgery, but subsequently shows a progressive decline, is an unusual feature, and Lecourtier & Kelly (2007) draw attention to the progressive alterations in serotonergic and GABAergic function within the IPN that occur after FR lesions (Takishita *et al*. 1990).

### (c) Aversive responses

The LHb is responsive to various noxious stimuli in the rat (Benabid & Jeaugey 1989) and the activation of LHb neurons by nociceptive inputs may be responsible for the inhibition of DA neurons of the SNc (Gao *et al*. 1996). In addition, induction of Fos immunoreactivity, which is indicative of neuronal activation, occurs in the LHb in response to stress (Smith *et al*. 1997) and both electrical stimulation or morphine injections into the habenula produces analgesia in a rat model of tonic pain (Cohen & Melzack 1985, 1986).

Several studies indicate that the DDC is involved in learning conditional avoidance responses (behavioural responses to avoid aversive stimuli). Habenular lesions appear to inhibit learning by reducing behavioural flexibility, especially under stressful conditions. For instance, in an operant one-way active avoidance task, Thornton & Bradbury (1989) found that habenular-lesioned rats were able to learn an escape response when the aversive stimulus (electroshock) was mild and the interstimulus time was long, but were defective, when compared with control animals, under more stressful conditions (higher stimulus intensities and shorter intervals between shocks). Furthermore, in a pre-pulse inhibition (PPI) task, designed to assess sensory gating and information filtering, mice with habenular lesions failed to show an increase in PPI after exposure to a fear-conditioning paradigm (Heldt & Ressler 2006). One explanation is that stress associated with fear-conditioning causes an increase in PPI in the wild-type mice, mediated by habenular effects on monoamine systems. Further support for a role of the DDC in adaptation to stress derives from the observation that plasma corticosterone levels are chronically elevated in FR-lesioned rats (Murphy *et al*. 1996). Recently, Pobbe & Zangrossi (2008) have investigated the involvement of the LHb in defence responses to stressful stimuli and shown opposite regulatory effects on escape behaviour (related to panic) versus inhibitory avoidance behaviour (related to generalized anxiety).

### (d) Circadian rhythms

The nuclei comprising the dorsal diencephalon are involved in regulating circadian rhythms. In addition to the habenulae, the epithalamus contains the pineal complex, and the pineal has a conserved role in the generation and/or regulation of circadian rhythms (for reviews, see Korf 1994; Falcon 1999). In lower vertebrates, the pineal is directly photoreceptive, enabling its circadian activity to be entrained to the 24 hour day–night cycle. In non-mammalian vertebrates, it comprises the clock, or pacemaker, of the circadian system and in all vertebrates is involved in the regulation of rhythmic behaviours and physiological responses through the secretion of melatonin (Falcon 1999). In mammals, the suprachiasmatic nuclei (SCN) of the hypothalamus act as the major pacemaker and receive light information via the retinohypothalamic pathway. The SCN clock controls the rhythmic activity of the pineal (Klein & Moore 1979) via the sympathetic nervous system and the pineal continues to modulate behaviour through the release of melatonin.

In addition to the pineal, the habenular complex appears to be involved in circadian functions. The LHb expresses melatonin receptors (Weaver *et al*. 1989) and in some species habenular cells synthesize melatonin (Sato *et al*. 1991). Hannibal (2002) identified numerous cell bodies, in both the MHb and LHb of the rat, which synthesize pituitary adenylate cyclase-activating peptide, which is also expressed in the retinohypothalamic tract and has been implicated in shifting the clock phase of the SCN (Harrington *et al*. 1999). Additionally, the LHb is innervated by SCN neurons (Buijs 1978) and melanopsin-expressing retinal ganglion cells (Hattar *et al*. 2006). Zhao & Rusak (2005) have shown that Hb neurons, especially in the LHb, respond to retinal illumination and show higher baseline firing *in vivo* during the day than the night. Moreover, LHb cells maintain this rhythmicity *in vitro* for at least 48 hours. While the functions of these oscillations and retinal illumination responses are unclear, accumulating evidence suggests that the habenular complex might form part of the output pathway regulating circadian rhythms that are generated in the SCN (e.g. Tavakoli-Nezhad & Schwartz 2006). Certainly, many of the behaviours influenced by the DDC show circadian variations, including sleep (below). Intriguingly, the LHb response to stress (assessed by c-Fos immunoreactivity) is greater during the night than the day (Chastrette *et al*. 1991).

### (e) Sleep

Evidence suggests that both the habenula and IPN are involved in regulating aspects of sleep. The habenula shows a significant increase in glucose usage during rapid eye movement (REM) sleep in cats (Lydic *et al*. 1991) and electrical stimulation of the LHb causes a decrease in REM sleep and an increase in non-REM sleep (Goldstein 1983).

Both the FR and IPN appear to be important regulators of normal sleep patterns and duration. Transecting the FR disrupts both the REM and non-REM components of sleep and hippocampal theta rhythms (Haun *et al*. 1992; Valjakka *et al*. 1998). Eckenrode *et al*. (1992) showed that transplants of a suspension of foetal habenular cells near the denervated IPN of FR-lesioned rats can restore normal patterns of substance P and/or choline acetyltransferase innervation. When substance P innervation of the IPN was restored, there was recovery of the integrity of REM sleep, whereas transplants that re-established cholinergic innervation restored the non-REM component (resulting in the recovery of sleep duration; Haun *et al*. 1992). Moreover, the extent of recovery was correlated with the number of transplanted cells. It was proposed that the function of the IPN in REM sleep might be mediated by its projection to the dorsal tegmentum, which in turn innervates the ‘REM sleep induction zone’ of the dorsomedial pons.

### (f) Reproductive and maternal behaviour

DDC circuitry appears to be involved in female sexual behaviour (Modianos *et al*. 1974). However, lesion studies have produced inconsistent results, showing either an increase or decrease in the receptivity of female rats after habenular lesions (discussed in Sutherland 1982). In male birds, the IPN displays changes in metabolic activity (as measured by increased deoxyglucose incorporation) during either appetitive or consummatory sexual behaviour (Dermon *et al*. 1999). Intriguingly, Kemali *et al*. (1990) observed that in the frog, the habenulae are larger in spring than in winter and this seasonal change in habenular size was most noticeable in females. As frogs are sexually active in spring, they hypothesized that hormonal signals initiating reproduction might mediate this effect on the habenula.

Maternal behaviour is also influenced by the DDC as evidenced by a reduction in pup retrieval, nursing and nest building following LHb lesions in rats (Matthews-Felton *et al*. 1995).

### (g) Involvement in psychosis

DDC circuitry is implicated in various psychological conditions including depression, anxiety, schizophrenia and neuropathological responses to addictive drugs.

In three animal models of depression, metabolic activity in the LHb is specifically increased (Caldecott-Hazard *et al*. 1988) and administration of the antidepressant drug tranylcypromine inhibits the elevation of metabolic rate in the LHb as well as the depressive behaviours. Furthermore, Thornton *et al*. (1985) found that habenular lesions blocked the effect of an antidepressant drug in reversing depressed behaviours that were induced in rats by forced swimming. The habenular complex provides the main forebrain projection to the raphe and exerts control over the activity of raphe neurons (Wang & Aghajanian 1977). LHb activity appears to inhibit raphe neurons as a result of activation of GABAergic interneurons (above). The midbrain raphe is the major source of cerebral serotonin and clinically effective antidepressants are thought to work by facilitating serotonergic signalling, suggesting that serotonin deficiency may underlie depression. Morris *et al*. (1999) observed that in human patients where depressive relapses were triggered by rapid depletion of blood tryptophan (the precursor to serotonin), positron emission tomography signals displayed correlated increases in the habenula and dorsal raphe as patients' rating of their depressed mood worsened. Recently, Yang *et al*. (2008) have reported that in two rat models of depression (where serotonin levels in the dorsal raphe are depleted), LHb lesions improved the behavioural responses of the rats and this was accompanied by an increase in the dorsal raphe serotonin levels. Overactivity of the LHb may therefore contribute to the pathogenesis of depression by inhibiting the raphe.

Pathology of the habenula may also contribute to schizophrenia (e.g. Lecourtier *et al*. 2004). In a human fMRI study, where subjects had to perform a difficult mental task and therefore made numerous errors, the habenular complex was activated when informative feedback was given about errors. Such activation did not occur in schizophrenic patients, who were also impaired in the task (Shepard *et al*. 2006). This result indicates that impaired activity of DDC circuitry is correlated with impaired cognitive performance in schizophrenia. Shepard *et al*. (2006) suggested that LHb dysfunction would limit a person's ability to learn from errors, which is one of the most characteristic cognitive deficits associated with schizophrenia. Although the cause and effects of the observed habenular dysfunction are unknown, there are some clues regarding potential pathological mechanisms. First, an elevated incidence of habenular and pineal calcification has been observed in schizophrenic patients (Sandyk 1992; Caputo *et al*. 1998). Second, influenza A virus, which increases the risk of schizophrenia if experienced prenatally, selectively attacks the habenula, paraventricular thalamic and brain stem monoaminergic areas when introduced into the mouse brain via the olfactory bulb (Mori *et al*. 1999).

DDC circuitry appears to be specifically vulnerable to the neurotoxic effects of addictive drugs (reviewed in Ellison 2002). Continuous administration of drugs that potentiate dopamine signalling, including cocaine, d-amphetamine and methamphetamine causes degeneration of axons in the sheath of the FR deriving from LHb neurons, whereas continuous nicotine causes a remarkably specific degeneration of axons from MHb neurons, which descend in the core of the FR. The FR may represent a ‘weak link’ that mediates the progressive effects of drug taking such as addiction and relapse and FR pathology may be involved in various psychoses (Ellison 2002). De Biasi & Salas (2008) have suggested that the effects of nicotine withdrawal are mediated by β4 nicotinic acetylcholine receptors in the MHb–IPN pathway.

## 3. Asymmetry within the epithalamus and DDC

A striking feature of the epithalamus is that its constituent nuclei display left–right (LR) asymmetries in many animal species. Indeed, structural asymmetries in this region have been described in virtually all classes of vertebrates (reviewed in detail by Concha & Wilson 2001). Asymmetries are most conspicuous in fishes, amphibians and reptiles, while birds and mammals show more subtle lateralization.

### (a) Habenular asymmetry

The habenulae display LR differences in size, cytoarchitectonic organization, neurochemistry and connectivity.

#### (i) Size

In the lamprey, the right habenular nucleus is considerably larger than the left (Yanez & Anadon 1994). This mode of lateralization is common in fishes; most actinopterygii (ray-finned bony fishes), with the exception of some teleost species, show rightward asymmetries in habenular size (see Concha & Wilson 2001).

In amphibians, the habenulae are divided into major dorsal and ventral nuclei, which are equivalent to the MHb and LHb of mammals, respectively (Harris *et al*. 1996; Guglielmotti & Fiorino 1999). Asymmetries have been described between the dorsal nuclei; the frog *Rana esculenta* shows a number of pronounced epithalamic asymmetries, including a larger dorsal nucleus on the left (Braitenberg & Kemali 1970; Kemali *et al*. 1990).

Habenular size asymmetries are considerably more subtle in birds and mammals. However, quantitative volumetric analyses have uncovered LR differences. Thus, in the albino rat, the left MHb is slightly (5%) larger than the right (Wree *et al*. 1981), whereas in the albino mouse rightward lateralization is apparent in the LHb during development and adulthood (Zilles *et al*. 1976).

#### (ii) Cytoarchitecture and cell morphology

Amphibians and reptiles show asymmetries in the subnuclear organization of the habenulae. Such asymmetries are also apparent in fish species, but are less conspicuous than the LR differences in size (see below; Signore *et al*. 2009).

In the enlarged right habenula of the lamprey, neurons are organized into three major layers, which are arranged dorsoventrally and separated by areas of neuropil, whereas only a single domain of periventricular neurons is seen on the left (Yanez & Anadon 1994).

In *R. esculenta*, the larger left dorsal habenula is subdivided into quite distinct medial and lateral subnuclei, whereas only a single nucleus comprises the right dorsal habenula (Gugliemotti & Fiorino 1998, 1999). In terms of both cytoarchitecture and cell morphology, the lateral subnucleus on the left is similar to the single right-sided nucleus. The left medial subnucleus possesses distinctive features and can be further subdivided into medial and lateral neuropils. It contains a unique population of large and ramified projection neurons that are absent from both the left lateral subnucleus and the right dorsal habenula.

#### (iii) Neurochemistry

Habenular lateralization is also manifest in terms of molecular differences between the left and right sides, including asymmetries in the distribution of neurotransmitters. For example, in the coho salmon, a discrete serotonin-immunoreactive subnucleus is found exclusively within the left habenula (Ekstrom & Ebbesson 1988).

The unique character of the medial subnucleus of the left dorsal habenula of the frog is further evidenced by its distinctive neurochemical properties. For instance, this subnucleus alone displays high levels of melatonin binding (Wiechmann & Wirsig-Wiechmann 1993) and calretinin immunoreactivity (Guglielmotti *et al*. 2004). Furthermore, NADPH-diaphorase histochemistry (which reports the presence of nitric oxide synthase in neural tissue; Hope *et al*. 1991) is exclusively localized within the lateral neuropil of the left medial subnucleus, but is not detected in the left lateral subnucleus nor the right dorsal habenula (Guglielmotti & Fiorino 1999).

#### (iv) Fibre tracts

LR differences between the habenulae are associated with asymmetries in the major efferent pathway from the dorsal diencephalon, the FR. Thus, in both the lamprey and the Siberian sturgeon, the larger right habenula is associated with a thicker right FR, and in the sturgeon, right-sided axons are larger in calibre than those on the left (Adrio *et al*. 2000).

Asymmetries in myelination have also been described. For instance, in the cartilaginous fish *Scyllium stellare*, only the larger left habenula contains neurons extending myelinated axons (Kemali *et al*. 1980; Miralto & Kemali 1980).

In addition to the asymmetrical subnuclear organization of the dorsal habenula in *R. esculenta*, the routing of axons towards the IPN is also asymmetric (Gugliemotti & Fiorino 1998). On the left side, the lateral subnucleus of the dorsal habenula gives rise to a tract that follows a peripheral route through the thalamus, whereas neurons of the medial subnucleus project axons along a more medial trajectory, bordering the third ventricle. These two contingents of the FR merge before innervating the IPN. On the right side, medial and lateral tracts are also formed, but they both derive from the single dorsal subnucleus of the right habenula.

As we discuss below, projections from the left and right habenulae also target different regions of the IPN in teleosts.

### (b) Pineal complex asymmetry

The pineal complex comprises the pineal, or epiphysis, and in some species a second nucleus, the parapineal. The pineal is likely to be present in all vertebrates and serves a neuroendocrine role, producing the hormone melatonin; in lower vertebrates, it is a photoreceptive structure (Falcon 1999; Concha & Wilson 2001). The pineal does not display overt asymmetry, being located at the dorsal midline of the epithalamus (although subtle asymmetries have been described in the location of the pineal stalk; Liang *et al*. 2000).

A second photoreceptive structure, the parapineal, or parietal eye, may also evaginate from the diencephalic roof plate. A parapineal has been described in lampreys, teleosts and certain species of lizard, but has not been detected in amphibians, birds or mammals. The parapineal consistently displays asymmetric connectivity within the epithalamus, projecting efferent axons that exclusively innervate the left habenula. In the species of lizards possessing a parietal eye,^1^ efferent axons innervate a restricted region of the left MHb (pars dorsolateralis; Engbretson *et al*. 1981). In teleosts, parapineal axons terminate in a defined rostrodorsal region of the left habenula (Concha *et al*. 2003; Signore *et al*. 2009); in the coho salmon, this terminal field may be coincident with the unilateral serotonergic subnucleus that is exclusively found in the left habenula (Ekstrom & Ebbesson 1988; Concha & Wilson 2001).

In lampreys and lizards, the parapineal/parietal eye is located at the dorsal midline, but in teleosts it is asymmetric both in its connectivity and location, being located entirely on the left side of the midline.

## 4. DDC asymmetries in zebrafish

The dorsal diencephalon and the associated circuitry of zebrafish have emerged as a useful model system for studying the development of neural lateralization (Concha 2004). Asymmetry phenotypes in the epithalamus emerge early in the development of zebrafish embryos—from one day post-fertilization—with very high reliability and consistent population laterality.^2^ Considerable progress has been made in understanding both the developmental pathways that produce asymmetry and that assign the laterality (direction or orientation) of those asymmetries. Moreover, *in vivo* analysis at the level of individual neurons has revealed a previously unrecognized mechanism by which neural circuitry on the left and right sides of the brain can be anatomically and functionally differentiated.

### (a) Lateralization in the zebrafish epithalamus

**Figure 2:**
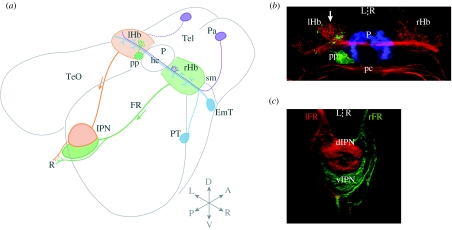
Asymmetric habenular circuitry in zebrafish. (*a*) Schematic showing connectivity of the habenular complex in larval zebrafish. A significant afferent input derives from migrated neurons of the eminentia thalami, which is thought to form the entopeduncular/peripeduncular complex in adult zebrafish (note that the teleostean entopeduncular complex is not part of the pallidum and does not correspond to the EP of amniotes; Wullimann & Mueller 2004). EmT neurons project bilaterally, innervating both left and right habenulae. A subset of left- and right-sided neurons in the anterior pallium are a source of asymmetric innervation, selectively terminating in a small medial domain of the right habenula (indicated in purple). In addition, a small afferent input may derive from the posterior tuberculum (Hendricks & Jesuthasan 2007). In the epithalamus, the left-sided parapineal exclusively innervates the left habenula (Concha *et al*. 2003). Habenular neurons project efferent axons that course in the fasciculus retroflexus. A major target is the interpeduncular nucleus: left- and right-sided axons are segregated along the dorso-ventral (DV) axis of the IPN in a *laterotopic* manner (Aizawa *et al*. 2005). A smaller and apparently symmetric contingent of habenular axons terminates caudal to the IPN in the serotonergic raphe. (*b*) Neuroanatomical asymmetries in the dorsal diencephalon. Anti-acetylated tubulin immunostaining (red) shows that the left habenula contains a greater density of neuropil, especially in the dorsomedial aspect of the nucleus. The pineal (blue) and parapineal (green) are visualized by the expression of green fluorescent protein (GFP) in a Tg(*foxD3*:GFP) transgenic larva. The parapineal is asymmetric in both its location and connectivity, and its efferent axons preferentially terminate in the asymmetric medial neuropil of the left habenula. Dorsal view, anterior top. (*c*) Three-dimensional confocal reconstruction showing habenular axon terminals in the ventral midbrain labelled using lipophilic tracer dyes applied to the habenulae. Left-sided axons were labelled with DiD (red) and right-sided axons with DiI (green). The dorsal IPN is almost exclusively innervated by left-sided axons, whereas the ventral target receives a majority of right-sided inputs. Dorsal view, anterior top. Tel, telencephalon; EmT, eminentia thalami; PT, posterior tuberculum; Pa, pallium; sm, stria medullaris; Hb, habenula; hc, habenular commissure; pp, parapineal, P, pineal; pc, posterior commissure; FR, fasciculus retroflexus; TeO, optic tectum; IPN, interpeduncular nucleus; a, anterior; p, posterior; l, left; r, right; d, dorsal; v, ventral. Adapted from Bianco *et al*. (2008). A number of these asymmetry phenotypes are conserved in the distantly related teleost medaka (*Oryzias latipes*; Signore *et al*. 2009).

By larval stages, several molecular and neuroanatomical asymmetries distinguish the left and right sides of the dorsal diencephalon (figure 2).

As in other teleosts, zebrafish possess a parapineal, which is asymmetrically located on the left side of the midline and exclusively innervates the left Hb (Concha *et al*. 2000, 2003; Signore *et al*. 2009). The parapineal has a bilateral origin: from approximately 28 hpf, precursor cells from the left and right sides of the midline in the anterior pineal anlage form a coherent group and begin to migrate towards the left Hb (Concha *et al*. 2003). This asymmetric migration represents one of the earliest signs of asymmetry in the dorsal diencephalon and is dependent upon fibroblast growth factor signalling. In zebrafish *fgf8* mutants, parapineal cells fail to migrate away from the dorsal midline (Regan *et al*. in press).

The larval habenulae also develop several asymmetric features, despite the fact that they display only modest LR differences in overall size (the left Hb is approx. 20% larger than the right; Halpern *et al*. 2003). The left habenula develops a greater density of neuropil than the right (Concha *et al*. 2000) and several molecular markers are expressed asymmetrically. Especially notable are the related genes, *leftover* (*lov*), *right-on* (*ron*) and *dexter* (*dex*), which are the members of the potassium channel tetramerization domain-containing family (Gamse *et al*. 2003, 2005). While *lov* is expressed more strongly on the left, *ron* and *dex* are expressed more extensively in the right Hb.

By adult stages, the dorsal region of the Hb contains discrete medial and lateral subnuclei that display distinct patterns of gene expression and efferent connectivity (Aizawa *et al*. 2005). The relative sizes of these subnuclei are LR asymmetric: in the left Hb, the lateral subnucleus is enlarged, whereas on the right side, the lateral subnucleus is small and the medial subnucleus contains the majority of neurons.

### (b) Asymmetric habenular circuitry

Recent studies have uncovered asymmetries in both the afferent and efferent connectivities of the larval zebrafish habenulae (figure 2).

A subset of pallial neurons, on both sides of the brain, project axons that innervate the habenulae asymmetrically and terminate within a small medial domain of the right habenula (Hendricks & Jesuthasan 2007).

Using lipophilic dye tracing to examine Hb efferent connectivity, Aizawa *et al*. (2005) showed that at larval stages, the Hb establishes strong projections to the IPN and anterior raphe. While the projection to the raphe appears symmetric, the origin of habenular axons innervating the IPN is conspiculously asymmetric. Left Hb axons preferentially terminate in a dorsal subdomain of the IPN (dIPN) and to a lesser degree in the ventral region of the IPN (vIPN), whereas almost all right habenular axons innervate the vIPN. This pattern of asymmetric connectivity is maintained at adult stages, at which time it is associated with a substantial asymmetry in the size ratios of habenular subnuclei: the adult left habenula contains a large lateral subnucleus,^3^ which innervates the dIPN, whereas in the right habenula, the lateral subnucleus is small and instead the medial subnucleus, which innervates the vIPN, is enlarged (Aizawa *et al*. 2005). To our knowledge, this represents one of the first examples in the vertebrates of LR differences in target connectivity for bilaterally paired neuronal nuclei. Because left and right axons become segregated along the dorso-ventral (DV) axis of their target, the Hb–IPN connectivity can be described as *laterotopic*.

The translation of LR asymmetry to a dorsoventral asymmetry potentially provides a mechanism to preserve LR coding in downstream circuitry. To address this hypothesis and determine whether distinct ‘left-derived’ and ‘right-derived’ circuits are maintained downstream of the IPN, future research should determine whether the dIPN and vIPN project to distinct efferent targets. Another aspect of the Hb–IPN connectivity pattern is that the convergence of Hb axons onto a unilateral midline target provides a means for lateralized, asymmetric neural processing in the epithalamus to modulate behaviours that require the regulation of bilateral motor circuitry on both sides of the midline.

#### (i) Lateralization in circuit microarchitecture

A recent study in our laboratory used focal electroporation to examine the morphology and connectivity of individual habenular projection neurons, which enabled the organization of the asymmetric Hb–IPN circuitry to be analysed at single-cell resolution (Bianco *et al*. 2008).

**Figure 3:**
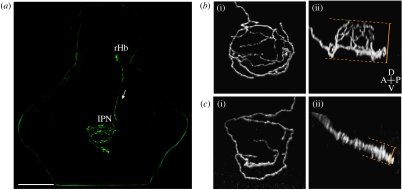
Zebrafish Hb–IPN projection neurons elaborate one of two distinct axon terminal arbour morphologies. (*a*) Three-dimensional reconstruction showing a single right habenular projection neuron that was labelled by focal electroporation with a construct driving the expression of membrane GFP, in an intact larval zebrafish brain (4 days post-fertilization). The cell body, located in the right habenula (rHb) extends an axon down the right FR (indicated by arrow) that terminates in the IPN. Habenular neurons elaborate remarkable axon arbours within the IPN that cross the ventral midline multiple times. Scale bar, 100 μm. (*b*(i),*c*(i)) Dorsal and (*b*(ii),*c*(ii)) lateral confocal reconstructions of single habenular axon arbours in the IPN. (*b*(i),(ii)) Example of an L-typical axon arbour, formed by 84% of left habenular neurons. These arbours are located in the dorsal IPN and are shaped similar to a domed crown and arborize over a considerable dorsoventral extent (compare dorsal (*b*(i)) and lateral (*b*(ii)) views of an example L-typical arbour). (*c*(i),(ii)) Example of an R-typical axon arbour, which is considerably flatter, localized to the ventral IPN and formed by 90% of right habenular neurons. Adapted from Bianco *et al*. (2008).

This approach led to the identification of two projection neuron subtypes that have axon terminal arbours with distinct morphologies and target connectivity (figure 3). Both subtypes are found in both the left and right habenula, but in substantially different ratios. Thus, the vast majority (84%) of left habenular neurons form ‘L-typical’ axon arbours that are tall and highly branched and localized to the dIPN. Only a very small percentage of the right-sided neurons form L-typical arbours. Instead, over 90 per cent of the right-sided cells elaborate ‘R-typical’ arbours that are flattened along the DV axis and localized to the vIPN. This arbour morphology is adopted by only a small number (16%) of left habenular neurons. Because these two arbour subtypes differentially innervate the dorsal and ventral domains of the IPN, the substantial asymmetry in the cell type composition between the left and right habenulae accounts for the *laterotopic* Hb–IPN connectivity pattern.

**Figure 4:**
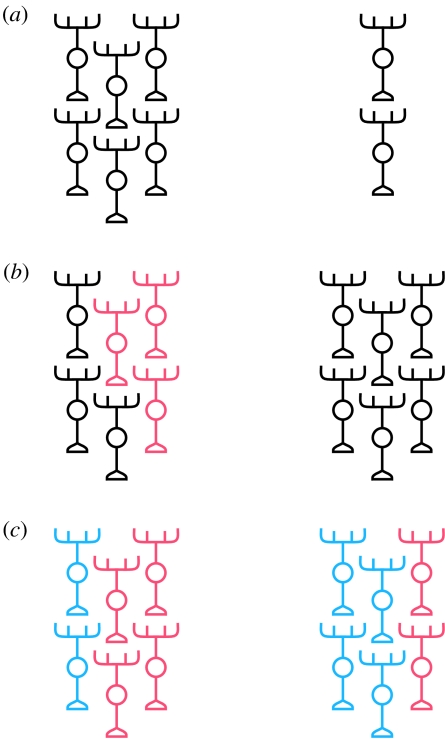
Models for lateralization of neural tissue. (*a*) Equivalent regions on the left and right of the CNS are identical in composition and differ only in overall size. (*b*) Unique types of neuron, or patterns of connectivity, may be specified on either the left or right or both sides (indicated by unique red neurons on the left in this schematic). (*c*) Identical circuit components might exist on both sides of the CNS, but in different ratios. Note that these models are in no way mutually exclusive. In fact, it is likely that all three strategies may be involved in the lateralization of DDC circuitry (see the main text).

This study has identified a fundamental strategy by which neural tissue on the left and right sides of the central nervous system (CNS) may become asymmetric. It gives rise to a model where the same or very similar circuitry components are produced on both sides, but in greatly different ratios, resulting in LR asymmetry in circuit microarchitecture that presumably translates into functional asymmetry. Figure 4 contrasts this model with two other models for how neural circuits might be lateralized. In perhaps the simplest model, equivalent regions on the left and right sides would contain the same classes of neuron and patterns of circuitry but differ only in size (figure 4*a*). As a result of such ‘scaling’, a particular cognitive function might be lateralized simply as a result of more neural substrate existing on one or the other side. In support of this possibility, Rosen (1996) observed that in the rat somatosensory/somatomotor cortex, asymmetry in tissue volume is strongly associated with the LR differences in the numbers of two subtypes of neuron, but there is only a weakly significant difference in cell packing density for one of the neuronal subtypes, suggesting that the left and right sides have similar neural architectures and show a proportional scaling to achieve differences in the quantity of neural tissue. In a third model, certain types of neuron, or patterns of connectivity, might be specific to one side and would not be present on the other side of the CNS (figure 4*b*). Hence, circuits on the left and right might receive different types of afferent inputs, perform different neural computations and/or connect to different downstream targets to mediate distinct types of cognition or behaviour. This mode of lateralization might be especially applicable to the zebrafish DDC: the parapineal projects exclusively to the left habenula and a subset of pallial neurons exclusively innervate a subdomain of the right habenula (above).

### (c) Development of circuit asymmetry

#### (i) Nodal signalling specifies the laterality of neural asymmetry

The discovery that components of the Nodal signalling pathway are expressed asymmetrically in the left dorsal diencephalon prior to the leftward migration of the parapineal and development of other lateralized phenotypes (Sampath *et al*. 1998; Concha *et al*. 2000; Liang *et al*. 2000) provided an entry point for the studies that have uncovered how neural asymmetry develops in the zebrafish DDC.

Work by Concha *et al*. (2000) and Gamse *et al*. (2003) established that the function of the left-sided Nodal signalling is to specify the direction, or laterality of asymmetry in the dorsal diencephalon. Over 95 per cent of wild-type larvae develop with a left-sided parapineal and left-sided elevation of *lov* expression; this strong population laterality requires unilateral, left-sided Nodal signalling. In experimental contexts where Nodal signalling is absent, or where it is activated bilaterally and therefore the asymmetry of its expression is lost, normal asymmetry phenotypes develop, but with randomized laterality: in 50 per cent of such embryos, the parapineal migrates to the left and the left Hb innervates the dIPN, but in the other 50 per cent the parapineal migrates to the right and the LR origin of axons in the dorsal and ventral IPN is perfectly reversed (Concha *et al*. 2000; Aizawa *et al*. 2005). Thus, Nodal signalling is not required for the development of neural asymmetry, but rather it appears to bias a stochastic laterality decision, to ensure consistent population laterality. Recently, Carl *et al*. (2007) and Inbal *et al*. (2007) have shown that proper regulation of Wnt/Axin/β-catenin signalling and functioning of Six3 proteins is required during gastrulation to repress Nodal signalling; this is an essential prerequisite to allow later unilateral activation of the pathway exclusively on the left side of the brain.

In addition to understanding how the laterality of neural asymmetries is specified, progress has been made in elucidating how asymmetry itself emerges in the DDC and resolving the developmental signalling mechanisms that control this process.

#### (ii) Asymmetric neurogenesis

Using BrdU birthdating to analyse the dynamics of Hb neurogenesis, Aizawa *et al*. (2007) provided an explanation for the substantially different ratios of distinct neuronal subtypes in the left and right habenulae.

The timing of neurogenesis was found to correlate with neuronal fate. Neurons born early tend to enter the LHb subnucleus. At adult stages, this subnucleus establishes connectivity exclusively with the dIPN and so it is very likely that these early-born neurons are L-typical, with basket-shaped, dorsally localized axonal arbours (see above). Later-born neurons tend to enter into the MHb subnucleus. As this connects to vIPN in adults, these later-born neurons are expected to comprise the R-typical subtype. Moreover, the time-course of neurogenesis was found to be LR asymmetric. On the left side, most neurons are born early, whereas on the right, neurogenesis is delayed and most neurons are born late. This is compatible with the observation that the majority of left-sided neurons acquire L-typical fate and the majority of right-sided neurons become R-typical (Bianco *et al*. 2008). The basis for this asymmetry in neurogenesis is as yet unknown.

#### (iii) Symmetry breaking and the parapineal

It has been suggested that the presence of a parapineal nucleus and the development of habenular lateralization might be causally associated (discussed in Engbretson *et al*. 1981; Harris *et al*. 1996; Concha & Wilson 2001; Guglielmotti & Cristino 2006). In species of lizard that possess a parietal eye, more pronounced asymmetries in habenular subnuclear organization are apparent than in species of reptiles lacking a parietal eye. However, in the lamprey, although the parapineal innervates the left habenula, it is the right nucleus that is enlarged. Moreover, striking habenular asymmetries have been described in vertebrates that appear not to possess a parapineal (e.g. amphibians), questioning any link between asymmetry in the habenulae and pineal complex.

Laser ablation studies in zebrafish have demonstrated that the parapineal is essential for the normal development of habenular lateralization. In larvae lacking a parapineal, both habenulae display patterns of gene expression and neuropil organization similar to those seen in the wild-type right habenula, and both sides project predominantly to the vIPN (Concha *et al*. 2003; Gamse *et al*. 2003, 2005; Bianco *et al*. 2008). However, subtle LR differences are retained in the molecular and neuroanatomical characteristics of the epithalamus (Concha *et al*. 2003; Bianco *et al*. 2008). Furthermore, despite the fact that they both target the vIPN, the axons of left- and right-sided habenular neurons retain distinct terminal morphologies (Bianco *et al*. 2008). This suggests that the parapineal does not specify LR identity in a binary manner, but rather it acts in concert with other, as yet unidentified, developmental signals to amplify LR asymmetry. These signals would then account for the maintenance of subtle lateralization following parapineal ablation.

The mechanism by which the parapineal acts and the molecular players involved in its signalling is as yet unknown. It is possible that the parapineal influences the time-course of Hb neurogenesis, possibly by modulating Notch signalling (the parapineal would be expected to suppress the Notch pathway on the left such that neurons are born early and acquire the L-typical/lateral subnucleus identity). However, the different terminal arbour morphologies of the left- and right-sided neurons in ablated larvae might be a result of interactions between axons that innervate the same target region (vIPN) at different times. In this model, the asymmetric time-course of Hb neurogenesis would be retained in parapineal-ablated larvae and the parapineal might contribute to Hb lateralization by promoting the proper differentiation of early-born left-sided neurons (including, for example, upreglation of guidance receptors that result in axon targeting to the dIPN; Kuan *et al*. 2007).

### (d) Asymmetric circuitry and asymmetric behaviour

What is the physiological and behavioural significance of the widespread asymmetries in the DDC? Although the answer to this question is unclear, behavioural analyses in zebrafish with alterations in DDC architecture and lateralization have the potential to help resolve the functions of this circuit and the importance of neural asymmetry.

Zebrafish display behavioural asymmetries in the form of biased turning direction and differential eye use for particular viewing tasks. This visual system lateralization takes a similar form to that in tetrapods where the right eye is used for examining complex or novel scenes and the left eye is used for viewing familiar objects (Miklosi *et al*. 1997; Miklosi & Andrew 1999). Moreover, a number of behavioural asymmetries are already apparent at larval stages (Watkins *et al*. 2004; Barth *et al*. 2005; Andrew *et al*. 2009).

Barth *et al*. (2005) examined both lateralized and non-lateralized behaviours in larvae and adults of the *frequent situs inversus* (*fsi*) line, in which a high frequency of fishes show concordant reversals in the laterality of both visceral (heart, pancreas, gut) and DDC asymmetries. While some asymmetric behaviours, including the pattern of left and right eye use in a mirror viewing task, are reversed in *fsi* fish with anatomical reversals, other lateralized behaviours do not reverse. Moreover, a novel, non-lateralized behaviour was observed in anatomically reversed larvae, which is not apparent in normally lateralized *fsi* fish or wild-types. These results suggest that there are multiple pathways specifying brain laterality, at least one of which is not concordant with visceral laterality and not affected in *fsi*. The emergence of novel behaviours might be a consequence of the erroneous superposition (or separation) of neural processing functions, arising from the reversals of a subset of neural asymmetries.

While the laterality of certain behaviours correlates with the reversals in the DDC, more work will be required to establish causal links between the activity and lateralization of this pathway and specific behavioural outputs. To this end, a variety of emerging technologies have the potential to be used in zebrafish: *in vivo* calcium imaging can be used to image activity in populations of neurons during animal behaviour (Niell & Smith 2005; McLean *et al*. 2007). Moreover, direct modulation of neural activity, for instance, using ectopically expressed proteins to excite or silence selected neurons (Knopfel 2008), should allow lateralized neurophysiology to be directly linked to specific behaviours.

## 5. Conclusion

In summary, the epithalamus constitutes the epicentre of the DDC. This highly conserved circuit is involved in a diverse range of behaviours, which we are still only beginning to understand. Neural asymmetries are present in this pathway in many species and range from LR differences in neurotransmitter expression to asymmetric patterns of connectivity. Several modes of lateralization can be seen in the DDC, including asymmetries in size and the production of unique circuitry components on one or the other side. Furthermore, large differences in the ratios of projection neuron subtypes generate LR asymmetric circuit microarchitectures. These neuronal subtypes are produced at different times during development, and asymmetries in the time-course of neurogenesis between the two sides of the epithalamus result in lateralized cell type compositions. Nodal signalling determines the laterality, or orientation, of such asymmetries. Signalling from the parapineal makes an important contribution to the development of asymmetry in the DDC of zebrafish, but other developmental signals must also be involved and the search for these continues.

## Endnotes

1 The parietal eye is considered an equivalent of the parapineal but forms a more sophisticated photoreceptive organ than is found in lampreys and teleosts. Its structure resembles the lateral eyes, and electrophysiological studies show it to be a fully functional photoreceptive organ; it emerges from a foramen in the skull.

2 If an asymmetry phenotype shows a consistent directionality in the majority of individuals in a population, it is said to show ‘directional asymmetry’ or ‘population laterality’. By contrast, if an asymmetric structure appears with equal probability on either the left or right side, there will be no consistent laterality within a population of animals, a situation referred to as ‘antisymmetry’.

3 The medial and lateral subnuclei described in the adult zebrafish habenula are not equivalent to the MHb and LHb of reptiles and mammals. Indeed, the entire teleost habenula is suggested to be homologous to only the MHb of reptiles/mammals (e.g. Yanez & Anadon 1996). However, further work is needed to determine homologies between habenular subnuclei among different classes of vertebrates.
